# Structural Engineering of Metal-Mesh Structure Applicable for Transparent Electrodes Fabricated by Self-Formable Cracked Template

**DOI:** 10.3390/nano7080214

**Published:** 2017-08-05

**Authors:** Yeong-gyu Kim, Young Jun Tak, Sung Pyo Park, Hee Jun Kim, Hyun Jae Kim

**Affiliations:** School of Electrical and Electronic Engineering, Yonsei University, 50 Yonsei-ro, Seodaemun-gu, Seoul 03722, Korea; y.g.kim@yonsei.ac.kr (Y.-g.K.); takyj88@yonsei.ac.kr (Y.J.T.); sppark@yonsei.ac.kr (S.P.P.); khjieu89@yonsei.ac.kr (H.J.K.)

**Keywords:** transparent electrode, metal-mesh, cracked template, colloidal silica

## Abstract

Flexible and transparent conducting electrodes are essential for future electronic devices. In this study, we successfully fabricated a highly-interconnected metal-mesh structure (MMS) using a self-formable cracked template. The template—fabricated from colloidal silica—can be easily formed and removed, presenting a simple and cost-effective way to construct a randomly and uniformly networked MMS. The structure of the MMS can be controlled by varying the spin-coating speed during the coating of the template solution or by stacking of metal-mesh layers. Through these techniques, the optical transparency and sheet resistance of the MMS can be designed for a specific purpose. A double-layered Al MMS showed high optical transparency (~80%) in the visible region, low sheet resistance (~20 Ω/sq), and good flexibility under bending test compared with a single-layered MMS, because of its highly-interconnected wire structure. Additionally, we identified the applicability of the MMS in the case of practical devices by applying it to electrodes of thin-film transistors (TFTs). The TFTs with MMS electrodes showed comparable electrical characteristics to those with conventional film-type electrodes. The cracked template can be used for the fabrication of a mesh structure consisting of any material, so it can be used for not only transparent electrodes, but also various applications such as solar cells, sensors, etc.

## 1. Introduction

A transparent conducting electrode is an essential component of a transparent device such as a display, touch screen, solar cell, sensor, or organic light-emitting diode. Such an electrode is generally made from a thin film composed of a layer of transparent conducting oxide—most commonly indium tin oxide (ITO). Thin films of this nature have excellent electrical characteristics and high transparency in the visible region. However, they are brittle and made from materials that are in short supply [1,2,3,4]. Moreover, they are known to cause device degradation due to a diffusion of metal atoms from ITO [5]. These critical drawbacks thus restrict their applicability. Recently, alternatives to these oxide materials have been reported, such as carbon nanotube (CNT) [6,7], graphene [8,9,10], conducting polymer [11,12], and metal nanowire [13,14]. Of these alternatives, metal nanowire is the most effective candidate for the replacement of ITO because it possesses the following advantages: low manufacturing cost, simplicity in large-area fabrication, and good stability [15]. However, a nanowire-based transparent electrode fabricated by the coating of pre-synthesized nanowires is limited in terms of its conductance, because of the contact resistance at junctions between the pre-synthesized nanowires [16,17].

Meanwhile, there have been some studies regarding the fabrication of wire structures based on the cracked template induced by physical stress [18,19]. However, it is difficult to apply the fabrication methods of these studies to a large area process due to a lack of uniformity and controllability of the structures. To overcome these issues, the use of TiO_2_ nanoparticles or polymer in the fabrication of a cracked template has been investigated [20,21]. The fabricated metal wires in these studies showed superior electrical conductance because of their well-organized interconnection and absence of junctions between metal wires. Thus, the fabrication of a wire structure based on a cracked template is a good way to construct networked metal wires with excellent electrical properties.

In this study, a cracked template formed by colloidal silica was used for the purpose of fabricating a highly-interconnected metal-mesh structure (MMS), and the characteristics of it were easily controlled by varying the coating condition of the template solution or by the stacking of metal-mesh layers. Colloidal silica is the suspension of silica nanoparticles in water, and is widely used as a binder [22], abrasive [23], catalyst [24], etc. The cracked template was formed by spin-coating colloidal silica onto a substrate and then allowing the substrate to dry, whereby the aggregation of silica particles during solvent evaporation occurs. An MMS was then successfully fabricated by depositing a metal layer onto the cracked template prior to removing the cracked template entirely. This structure exhibited a randomly-interconnected network, and enabled the fabrication of highly transparent and conducting electrodes. Furthermore, flexibility tests of the MMS were conducted, and MMS electrodes were applied to In-Ga-Zn-O (IGZO) thin-film transistors (TFTs) to verify the applicability of the MMS for flexible electronics and real devices, respectively.

## 2. Results and Discussion

**Figure 1:**
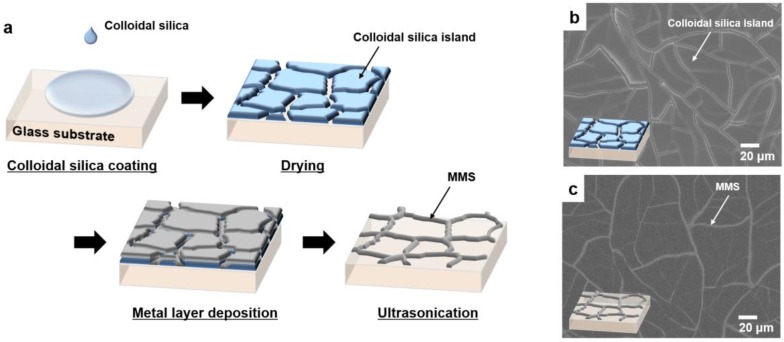
(**a**) Schematic diagram of the fabrication process for a metal-mesh structure (MMS) using a cracked template; scanning electron microscopy (SEM) images of (**b**) colloidal silica layer on the substrate after drying; and (**c**) fabricated MMS. Insets in (b) and (c) show the schematic diagram of each sample.

During the drying process upon the coating of colloidal silica on a substrate, silica nanoparticles aggregated due to the solvent evaporation, and island structures were formed as shown in Figure 1b. Figure S1a shows a cross-sectional scanning electron microscopy (SEM) image of such an island structure with a thickness of ~800 nm. The island structure is comprised of physically adsorbed silica nanoparticles. Therefore, it can be easily removed by physical treatments such as ultrasonication. We identified that the silica island layer was clearly removed after ultrasonication in water, as shown in Figure S1b. Thus, the colloidal silica-based island structures which can be easily formed and removed were suitable for a cracked template. Upon the deposition of a metal layer and the removal of the cracked template, only metals deposited on the cracks remained; all metals on island structures were removed with the cracked template during the ultrasonication process. As a result, an MMS-shaped randomly networked mesh structure corresponding to the cracks of the cracked template was fabricated (see Figure 1c).

**Figure 2:**
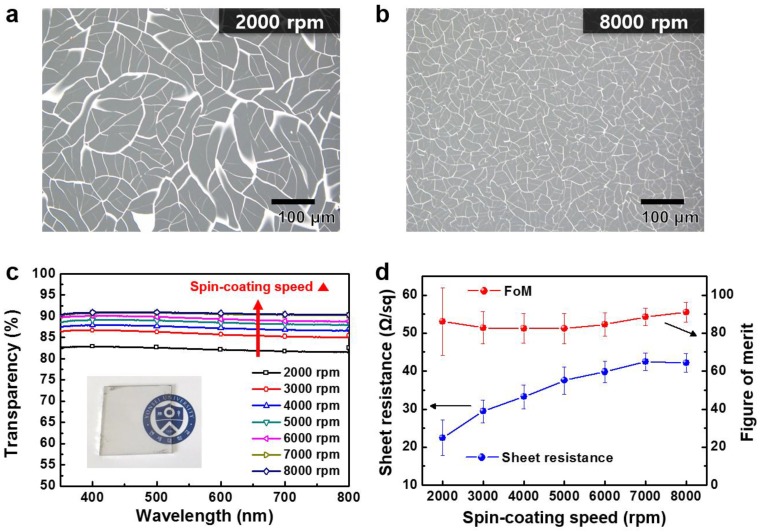
Optical microscopy (OM) images of the fabricated MMS using different spin-coating speeds; (**a**) 2000 and (**b**) 8000 rpm; (**c**) optical transparency in the visible region; and (**d**) sheet resistance and figure of merit (FoM) of the fabricated MMS using different spin-coating speeds. Inset in (b) shows a photograph of the MMS on a glass substrate (2.5 cm × 2.5 cm) fabricated with a spin-coating speed of 7000 rpm.

The size of colloidal silica-based island structures pertaining to the cracked template can be controlled by varying the spin-coating speed. Figure 2a,b show optical microscopy (OM) images of the MMS fabricated by a cracked template method that involved the use of different spin-coating speeds for the coating of the cracked template solution. For a cracked template fabricated under a low spin-coating speed (i.e., the coated solution was thick), particles in colloidal silica solution form the islands by aggregation with farther particles, resulting in larger islands and wider cracks compared with the one fabricated under a higher spin-coating speed [25,26]. Consequently, an MMS fabricated by a cracked template formed with a low spin-coating speed will consist of wider and sparser wires compared with that using a cracked template formed with a high spin-coating speed (which will consist of narrower and denser wires). However, a cracked template fabricated under a spin-coating speed of less than 2000 rpm is not able to endure damage caused by sputtering and will thus break at the stage of metal deposition. Hence, it is only possible to form an MMS using a cracked template that has been fabricated under a spin-coating speed of more than 2000 rpm, as shown in Figure S2a.

Figure 2c shows the optical transparency in the visible region as a function of wavelength, measured using an ultraviolet (UV)-visible spectrophotometer. As the spin-coating speed was increased, a higher optical transparency was obtained owing to a decrease in the width of metal wires. All the MMS samples showed an optical transparency above 80% in the visible region, and the MMS fabricated with a spin-coating speed of more than 6000 rpm showed a high level of transparency (>90%).

To verify the electrical characteristics of the fabricated MMSs, a sheet resistance measurement was conducted. It can be seen that the sheet resistance increases with spin-coating speed, as shown in Figure 2d. This phenomenon corresponds with the results of optical transparency measurement, which showed trade-off relationship between optical transparency and sheet resistance [27,28]. This is attributable to relatively wide cracks, which occur when low spin-coating speeds are used; such cracks lead to the formation of wide metal wires. These wide cracks in the template resulted in a decrease in optical transparency and an increase in electrical conductance. Regardless of the spin-coating speed, the MMS showed significantly low sheet resistance with high optical transparency, and these results were attributable to well-interconnected metal wires without junctions between the metal wires. The characteristics of the MMSs in this study are comparable or superior to those of previous studies investigating Al-based mesh structures [29,30].

To investigate the characteristics of an MMS to be used as a transparent electrode, figure of merit (FoM), which has been widely used to judge the performance of a transparent electrode, was calculated. FoM is defined as the ratio of the electrical to optical conductivity and can be calculated by the following equation [31,32,33]:

$$\mathrm{FoM}=\frac{\sigma_{dc}}{\sigma_{opt}}=\frac{188.5}{R_{s}(\frac{1}{\sqrt{T}}-1)}$$

where *σ_dc_*, *σ_opt_*, *R_s_*, and *T* are the electrical conductance, optical conductance, sheet resistance, and transparency at 550 nm, respectively. The calculated FoM values are shown in Figure 2d; they do not vary significantly with the spin-coating speed. Therefore, by changing the spin-coating speed, both the optical transparency and the sheet resistance can be designed without the degradation of FoM.

In addition, we identified the effects of the drying temperature of colloidal silica on the optical and electrical characteristics of the MMSs. The drying process was conducted at room temperature, 100, 200, and 300 °C for 5 min. The optical transparency and sheet resistance values for the fabricated MMSs under the different drying temperatures are shown in Figure S3. In this case, the spin-coating speed was fixed at 7000 rpm for all the samples. The optical and electrical characteristics of the MMSs were not significantly affected by drying temperature. The cracked templates in each case were fabricated not by the chemical reaction but by the physical aggregation of silica particles, so the temperature during the drying process did not affect the structure of the MMS.

**Figure 3:**
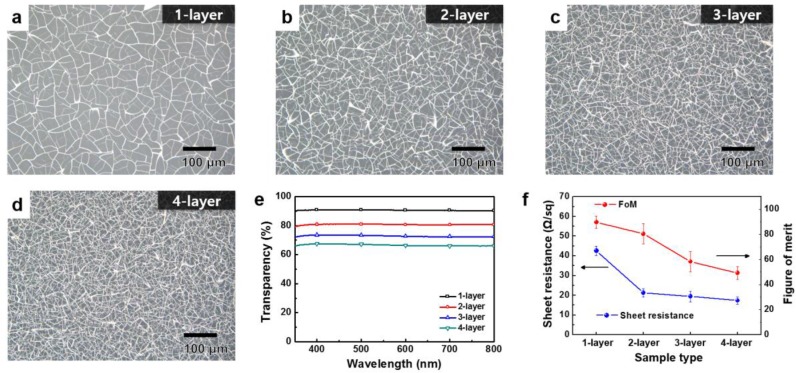
OM images of the multi-layered MMSs; (**a**) single-; (**b**) double-; (**c**) triple-; and (**d**) quadruple-layer; (**e**) optical transparency in the visible region; and (**f**) sheet resistance and calculated FoM of the fabricated multi-layered MMSs.

Furthermore, the structure of metal-mesh was controlled by multi-stacking the MMS. Figure 3a–d show OM images of multi-layered MMSs fabricated under a spin-coating speed of 7000 rpm. As the number of stacked layers increased, the corresponding MMSs were found to be denser and more interconnected to each other. These structural changes coincided with the electrical and optical characteristics shown in Figure 3e,f. Multi-stacking of MMS is directly related to a decrease in optical transparency and sheet resistance. For a double-layered MMS, even if the optical transparency was decreased, it still showed a high optical transparency of approximately 80% in the visible region. In addition, it showed a significant decrease in sheet resistance (~20 Ω/sq) compared with a single-layered MMS (~40 Ω/sq). In the case of the triple- and quadruple-layered MMS, the optical transparency decreased with stacking the layer, but the decrease in sheet resistance was not significant. This can be explained by the notion that the third or fourth stacked layers performed nothing more than a subsidiary role based on the pre-fabricated double-layered MMS in terms of the electrical conduction, because the networking of wires was sufficiently well-organized to have high electrical conductivity when the second MMS layer was stacked. These results were also reflected in the corresponding FoM values. FoM was significantly decreased when a third MMS layer was deposited, as shown in Figure 3f.

**Figure 4:**
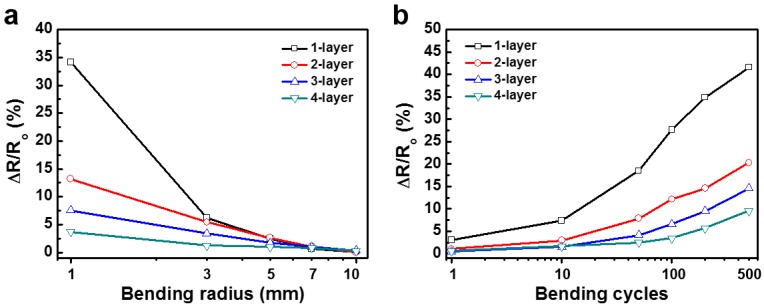
Variations in the resistance of single- or multi-layered MMSs on a flexible substrate as a function of (**a**) bending radius; and (**b**) the number of cycles repeated under bending radius of 1 mm.

We conducted bending tests on the single-, double-, triple-, and quadruple-layered MMSs to verify the flexibility of each MMS. Figure 4a shows the percentage of resistance variation as a function of bending radius. The resistance variation decreased with the stacking of additional MMS layers, meaning that the flexibility increased with the stacking of additional MMSs. This is because a multi-layered MMS has a more highly networked wiring structure than a single-layered one. Therefore, even if some wires are disconnected during bending, other wires can effectively compensate for the disconnection. The bending endurance tests were also conducted by repeating the bending with a bending radius of 1 mm, the results of which are shown in Figure 4b. Identical to the results of the flexibility test under different bending radii, the stacking of multiple MMS layers can improve the bending endurance of an electrode. As a result, the more MMS layers were stacked, the better flexibility was obtained. Therefore, a multi-layered MMS is more suitable for flexible applications than a single-layered MMS.

**Figure 5:**
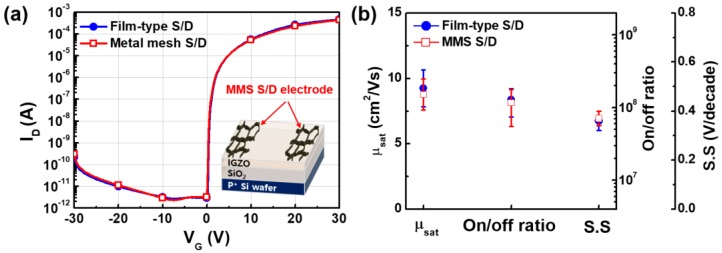
(**a**) Transfer characteristics; and (**b**) electrical parameters of In-Ga-Zn-O (IGZO) TFTs using conventional film-type or MMS source/drain (S/D) electrodes with error bars.

Additionally, to ascertain the applicability of an MMS in the case of practical devices, it was applied to the source/drain (S/D) electrodes of an IGZO TFT. Figure 5a shows the transfer characteristics of IGZO TFTs with conventional film-type and MMS S/D electrodes. The transfer characteristics were measured with a drain voltage (V_D_) of 20 V, and TFTs with two kinds of electrodes showed similar transfer characteristics. The electrical parameters, including saturation field-effect mobility (μ_SAT_), on-to-off current ratio (on/off ratio), and subthreshold swing (S.S), of 18 IGZO TFTs fabricated with film-type S/D electrodes and MMS S/D electrodes are shown in Figure 5b, with error bars. The electrical parameters were extracted from the transfer characteristics of TFTs based on previous studies [34,35]. As shown in Figure 5, the TFTs with MMS S/D electrodes showed comparable electrical characteristics and good uniformity to those with film-type S/D electrodes; this reveals that an MMS can be applied to a practical device with high uniformity and without significant degradation of electrical performance.

## 3. Materials and Methods

### 3.1. Fabrication of Networked MMS

An MMS based on a lift-off process using a cracked template was fabricated, the experimental procedure for which is illustrated in Figure 1. After cleaning the substrate, a deep UV light (185 nm and 254 nm) sourced by a mercury lamp was irradiated to the substrate in air to make a hydrophilic surface. Then, colloidal silica was spin-coated onto the UV-treated substrate at various coating speeds (from 1000 to 8000 rpm, at intervals of 1000 rpm) for 30 s. The colloidal silica used in this study was a suspension of 12-nm-sized silica particles with 30 wt % in water. The cracked template was fabricated by drying the solvent of colloidal silica in air at room temperature for 5 min. After drying, 150 nm of Al metal layer was deposited on the cracked colloidal silica layers by radio frequency (RF) magnetron sputtering. Finally, to remove the cracked template, ultrasonication was conducted in water for 5 min. In the case of the multi-layered MMS, the procedure described above was repeated several times over.

### 3.2. Fabrication of IGZO TFTs with MMS Electrodes

IGZO TFTs with an inverted staggered structure were fabricated. A heavily boron-doped p-type Si wafer with thermally oxidized SiO_2_ of 1200 Å was used as a substrate. The p^+^ Si wafer and SiO_2_ layer were used as a gate electrode and a gate dielectric, respectively. An IGZO active layer of 40 nm was deposited by RF magnetron sputtering on a cleaned substrate, and was annealed at 300 °C for 1 h. To make a cracked template, the colloidal silica solution was coated on the IGZO layer at a spin-coating speed of 7000 rpm. After drying at room temperature for 5 min, 80-nm-thick Al used as S/D electrodes was deposited by RF magnetron sputtering via a shadow mask. Finally, ultrasonication in water was conducted to remove the cracked template. The width and length of the channel were 1000 and 150 mm, respectively.

## 4. Conclusions

We successfully fabricated an MMS applicable for transparent and flexible electrodes by using a cracked template that is easily formed and removed. This represents a cost-effective way to fabricate a networked MMS with high uniformity. The structural control of MMS was developed by varying the coating condition or by the stacking of multiple MMS layers. As the spin-coating speed was decreased or the number of stacked layers was increased, both the optical transparency and the sheet resistance of an MMS decreased. The double-layered Al MMS fabricated with a spin-coating speed of 7000 rpm showed high optical transparency (~80%) in the visible region, low sheet resistance (~20 Ω/sq), and small resistance variation during the bending test compared with a single-layered MMS. In addition, an MMS was applied to S/D electrodes for IGZO TFTs, and the TFTs with MMS electrodes showed comparable characteristics to that of the TFTs with conventional film-type electrodes. Therefore, an MMS fabricated using a colloidal silica-based cracked template can be applicable for transparent and flexible devices. The cracked template based on the colloidal silica can be used for the fabrication of mesh structures, regardless of the material to be used. Hence, it can be applicable to not only transparent and flexible electronics, but also to various applications where a networked mesh structure is required.

## Supplementary Materials

The following are available online at http://www.mdpi.com/2079-4991/7/8/214/s1, Figure S1: (a) Cross-sectional SEM image of colloidal silica layer after drying; (b) SEM image of the cracked template fabricated by colloidal silica and the substrate surface after removal of the cracked template using ultrasonication, Figure S2: OM images of the fabricated NM using different spin-coating speeds: (a) 1000; (b) 2000; (c) 3000; (d) 4000; (e) 5000; (f) 6000; (g) 7000; and (h) 8000 rpm, Figure S3: (a) Optical transparency in the visible region and (b) sheet resistance of the fabricated MMS using different drying temperatures.
