# Supplementary material for: Structural Engineering of Metal-Mesh Structure Applicable for Transparent Electrodes Fabricated by Self-Formable Cracked Template
Source: Nanomaterials (Basel). 2017 Aug 5;7(8):214. doi: 10.3390/nano7080214 (PMC5575696; doi:10.3390/nano7080214)
Supplement: Supplementary file 1 [file nanomaterials-07-00214-s001.pdf]

Supplementary Materials

Structural engineering of metal-mesh structure applicable for transparent electrode fabricated by self-formable cracked template

Yeong-gyu Kim, Young Jun Tak, Sung Pyo Park, Hee Jun Kim, and Hyun Jae Kim*

School of Electrical and Electronic Engineering, Yonsei University, 50 Yonsei-ro, Seodaemun-gu, Seoul, 03722, Republic of Korea

*Corresponding author: Hyun Jae Kim (hjk3@yonsei.ac.kr)

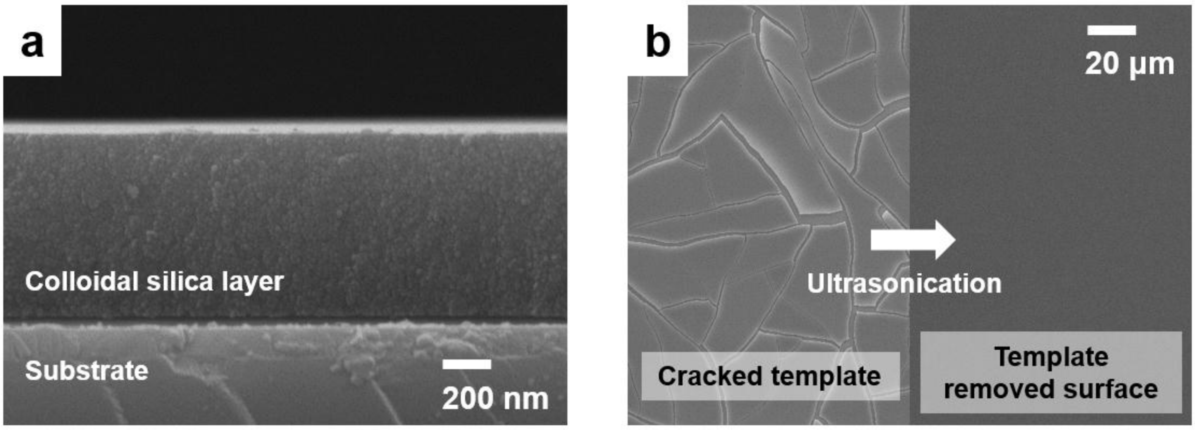

Figure S1. (**a**) Cross-sectional SEM image of colloidal silica layer after drying, (**b**) SEM image of the cracked template fabricated by colloidal silica (left) and the substrate surface after removal of the cracked template using ultrasonication (right).

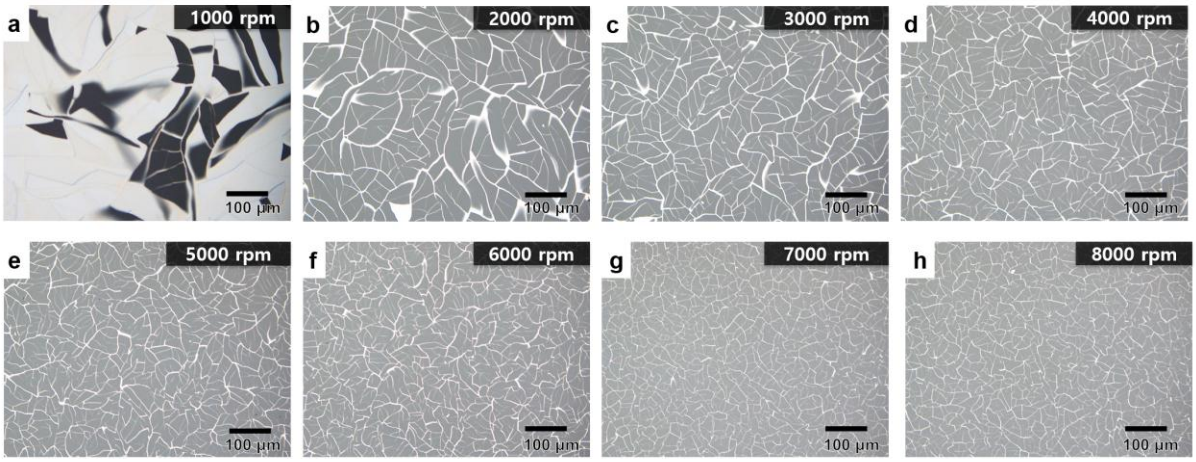

Figure S2. OM images of the fabricated NM using different spin-coating speeds; (**a**) 1,000 and (**b**) 2,000, (**c**) 3,000, (**d**) 4,000, (**e**) 5,000, (**f**) 6,000, (**g**) 7,000 and (**h**) 8,000 rpm.

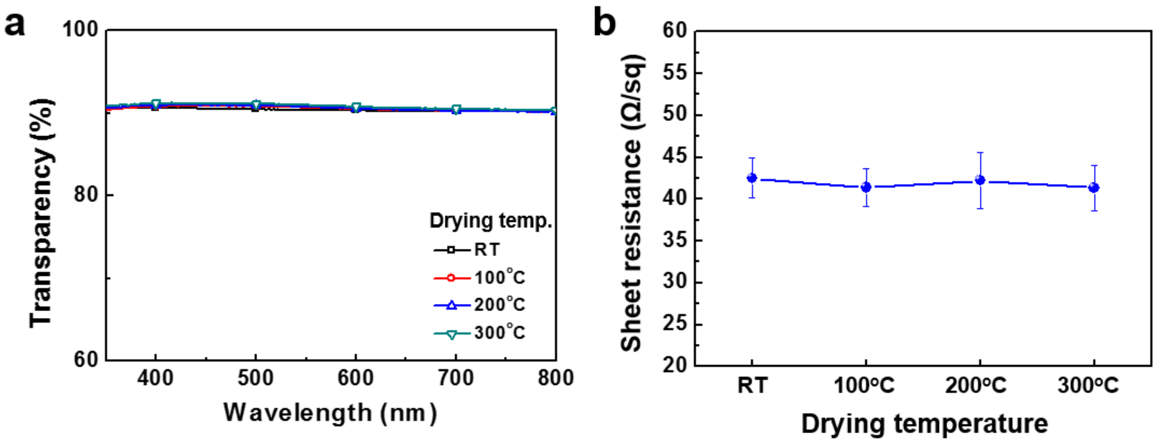

Figure S3. (**a**) Optical transparency in the visible region and (**b**) sheet resistance of the fabricated MMS using different drying temperature.
